# Rebleeding of a Splenic Artery Aneurysm after Coil Embolisation

**DOI:** 10.1155/2016/1858461

**Published:** 2016-10-31

**Authors:** Kyra D. Kingma, Antonius N. van der Linden, Rudi M. H. Roumen

**Affiliations:** ^1^Emergency Department, Máxima Medical Centre, Veldhoven, Netherlands; ^2^Radiology Department, Máxima Medical Centre, Veldhoven, Netherlands; ^3^Surgery Department, Máxima Medical Centre, Veldhoven, Netherlands

## Abstract

*Background*. Splenic artery aneurysm (SAA) is an uncommon and difficult diagnosis. SAA is more common in females. Only 20% of SAA is symptomatic and may present as a rupture. A ruptured SAA is associated with a 25% mortality rate.* Case Presentation*. We present a case of a male patient with a bleeding SAA that rapidly increased in size. Distal coiling was technically impossible and despite proximal coil embolisation the SAA continued to bleed. A laparotomy including splenectomy and partial pancreatectomy was performed with an uneventful patient recovery.* Discussion*. Endovascular management is currently considered the optimal treatment of SAA. However, careful monitoring and follow-up is needed after embolisation as rapid recanalization of the SAA may possibly occur, especially when distal coiling of the aneurysm is unsuccessful.* Conclusion*. Endovascular treatment of an SAA is not necessarily effective. Surgeons must be prepared to perform open procedures to further reduce mortality rates.

## 1. Introduction

Splenic artery aneurysm (SAA) is defined as a more than 1 cm dilatation of the splenic artery diameter [1]. The incidence varies from 0,05% to as high as 10,4% in postmortem studies [2, 3]. An SAA is four times more common in females [4, 5]. The pathogenesis is not completely understood, but risk factors include pregnancy, trauma, portal hypertension, arterial degeneration, and atherosclerosis [1, 6]. 80% of splenic artery aneurysms are asymptomatic [7]. If symptoms are present, however, abdominal pain in the epigastrium or left upper quadrant is reported. Other symptoms may include anorexia, nausea, or vomiting. Spontaneous rupture is found in 2–10% of patients on initial presentation and is associated with an overall 25% mortality [8]. A 95% rupture rate in pregnancy is reported with a dreadful 75% mortality rate [7, 9]. The preferred treatment method at present is endovascular embolisation of the splenic artery. This technique is considered safe and less invasive than open surgery [1].

We describe a rare case of a ruptured splenic artery aneurysm in a male patient that continued to bleed despite embolisation of the proximal splenic artery.

## 2. Case Presentation

**Figure 1:**
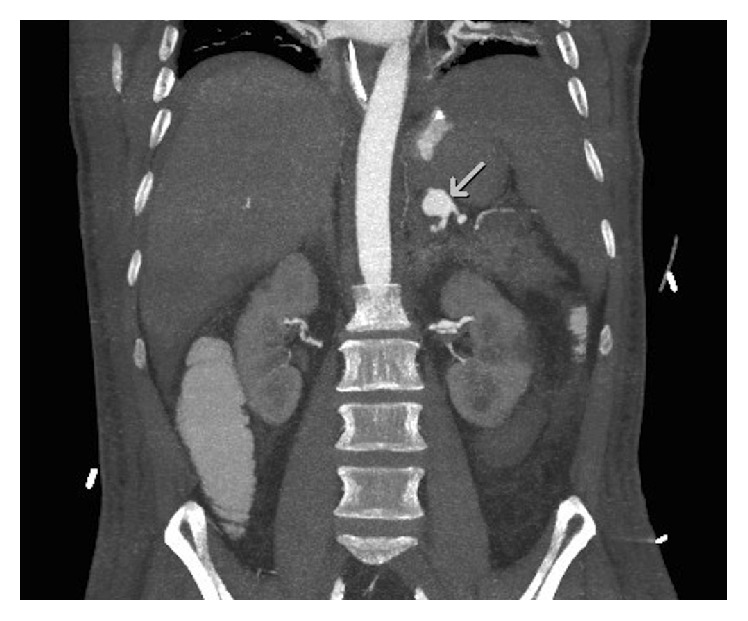
Splenic artery aneurysm (arrow) of 14 mm on abdominal computed tomographic angiography.

**Figure 2:**
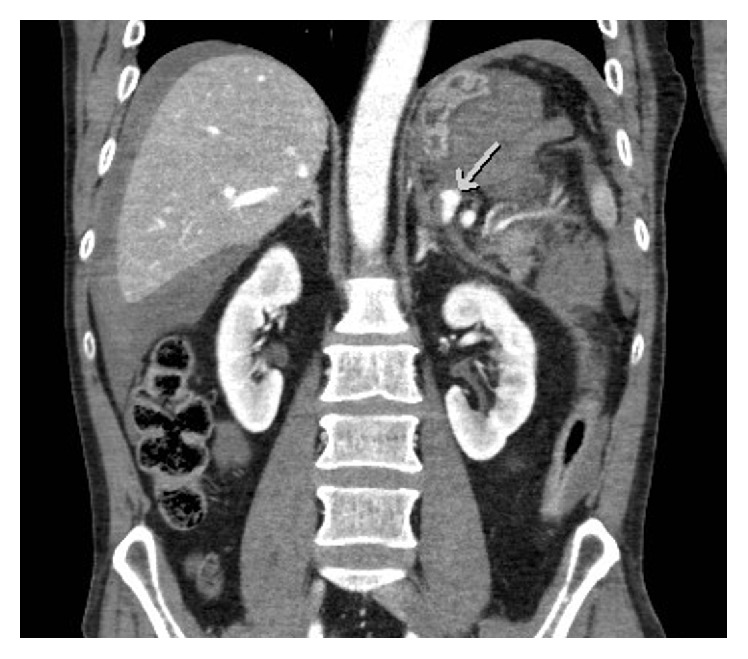
9 mm contrast-filled dilatation of splenic artery (arrow) on initial CT.

A 59-year-old man with hypertension and a family history that included two brothers who died of an aortic aneurysm was brought to the Emergency Department after falling from his bicycle. He reported abdominal pain since one day. On examination, he was pale and had a pulse rate of 64/min, a blood pressure of 70/50 mmHg, a body temperature of 36°C, and a respiratory rate of 16 breaths/min. He had abdominal tenderness with muscular rigidity, especially in the upper abdominal region. A full blood count showed a haemoglobin level of 6,4 mmol/L, while all other laboratory tests were within the normal range. A chest X-ray showed normal lung fields and no pneumoperitoneum. As a vascular incident was feared, an abdominal ultrasound revealed free abdominal fluid around the liver and spleen without an aortic aneurysm. An abdominal computed tomographic (CT) demonstrated fluids in the omental bursa with a normal aspect of the pancreas but was unable to point to the origin of the free fluid. After resuscitation using standard techniques, the patient was transferred to the operating room under the diagnosis of intra-abdominal haemorrhage of unknown origin. During laparotomy, three litres of blood were obtained from the abdominal cavity but no active bleeding was found. Inspection of the omental bursa revealed a hematoma surrounding the pancreas. The patient was packed using several large volume gauzes and admitted to the intensive care unit. The next day, these gauzes were removed and again no active bleeding was seen. The next four days were uneventful. However, on the fifth day, the patient developed acute severe abdominal pain. An abdominal computed tomographic angiography (CTA) revealed a 14 mm splenic artery aneurysm (Figure 1) as well as a 12 mm aneurysm of the coeliac trunk. In retrospect, on the first CT, a 9 mm focal contrast-filled dilatation of the splenic artery could already have been identified in a large hematoma (Figure 2). This was initially missed.

**Figure 3:**
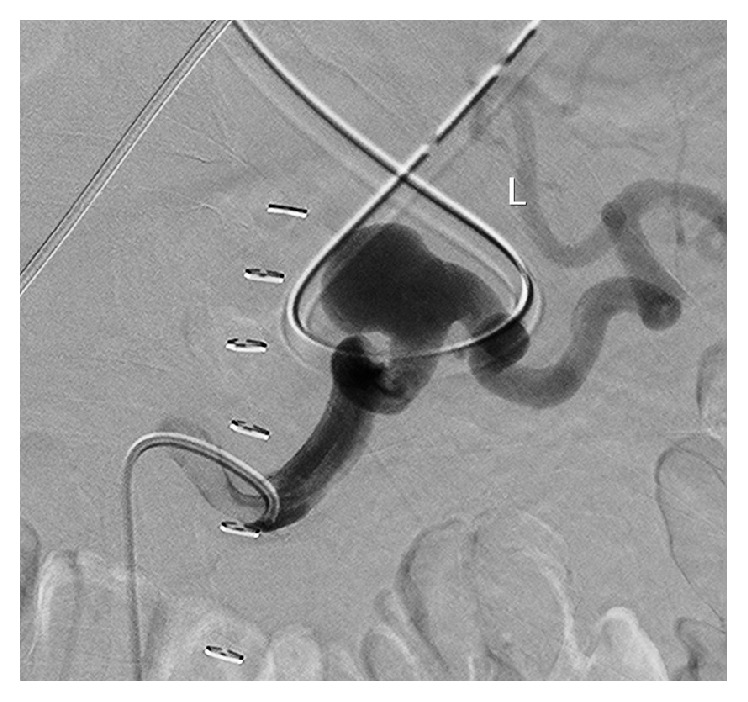
Splenic artery aneurysm with a tortuous aspect during embolisation procedure.

A ruptured splenic aneurysm was now considered the source of the abdominal bleeding and the patient underwent a coil embolisation of the proximal splenic artery using a femoral approach (Figure 3). The splenic artery appeared very delicate and reacted with intense vasospasm following minimal movement of the endovascular catheter tip. Fear of arterial dissection together with the tortuous aspect of the distal splenic artery urged the interventionalist to refrain from coil embolisation at the distal end of the aneurysm. Flow in the distal part of the splenic artery was absent after placement of 8 coils proximal to the aneurysm. During the endovascular procedure, it was noted that the aneurysm had grown further to 18 mm. Although initially stable, some 24 hours after the embolisation, the patient again experienced severe abdominal pain and became hemodynamically instable. After cardiopulmonary resuscitation, a CTA was repeated and revealed recanalization of the splenic artery without migration of the coils. An urgent laparotomy was performed with removal of the spleen and the tail of the pancreas, encountering the ruptured aneurysm. The patient recovered uneventfully and was discharged from the hospital 7 days later. He will undergo vaccination and follow-up for his coeliac trunk aneurysm.

## 3. Discussion

Our case illustrates the difficulty of diagnosing a splenic artery aneurysm. Most splenic artery aneurysms are asymptomatic, but even when patients have symptoms, diagnosis is challenging. Our initial CT demonstrated a 9 mm splenic artery diameter which is slightly enlarged but not an aneurysm yet. The patient's splenic artery dilated fast during the next days. The literature states that all asymptomatic aneurysms greater than 2 cm in diameter and those increasing in size regardless of size should be treated, as should symptomatic splenic artery aneurysms and those during pregnancy [1].

Pseudoaneurysms have a higher risk of rupture compared to true aneurysms and should therefore be treated as soon as possible [10–12]. Pseudoaneurysms are defined as expansions of the artery with focal disruption of the arterial wall, whereas true aneurysms are defined as expansions of all wall layers [13]. Pseudoaneurysms can be caused by trauma and iatrogenic lesions or from inflammatory of infectious conditions [14–16]. Our patient had suffered from abdominal pain before he fell from his bicycle. Therefore, we found a true aneurysm more likely than a traumatic pseudoaneurysm.

There are various therapeutic options available but a transcatheter embolisation is currently considered the standard of care. This technique is preferred to surgery, especially when the aneurysm is difficult to manage surgically and in high-risk patients. Two techniques are advocated. The packing technique is used to embolise the aneurysm itself. The isolation technique is achieved by occlusion of both the proximal and distal feeding arteries to the aneurysm to prevent backbleeding into the lesion [17, 18]. In our case, the isolation technique was preferred, but distal coiling was technically impossible. Because the aneurysm could not be passed with the guide wire, stenting the aneurysm was also impossible. In the acute situation, we tried to stop the bleeding with proximal embolisation. After coil placement at the proximal end flow through the aneurysm had ceased and the procedure was presumed successful. However, swift recanalization occurred, possibly because of retrograde collateral filling. In retrospect, the use of a micro catheter might have allowed for cannulating the distal end of the aneurysm without spasm or dissection.

Technical success of coil embolisation of visceral artery aneurysms varies from 67 to 92% in published data [19–21]. Risk of recanalization depends on location, nature of the aneurysm, and embolisation technique used. A 5% reperfusion rate in visceral (pseudo)aneurysms is reported [10]. After embolisation of a splenic artery aneurysm, a 12,5% partial recanalization rate is described [17].

No previous cases were described in which only proximal coiling was performed.

Our case suggests a very high risk of recanalization and bleeding after incomplete coiling. Therefore, if this is the case, patients should be monitored very closely so that immediate surgical intervention can be instituted when patients demonstrate signs of rebleeding.

## 4. Conclusion

Splenic artery aneurysms are often asymptomatic and rare and therefore difficult to diagnose. When a patient presents with left upper quadrant or epigastric abdominal pain and signs of haemorrhage or shock, SAA should be considered. Embolisation is the first line of treatment. Recanalization with haemorrhagic shock can occur, particularly after an incomplete embolisation. Therefore, a close monitoring after embolisation is needed. If rebleeding is probable, open surgery can be lifesaving.
